# Supplementary material for: Evaluation of African Swine Fever Virus E111R Gene on Viral Replication and Porcine Virulence
Source: Viruses. 2023 Mar 30;15(4):890. doi: 10.3390/v15040890 (PMC10143872; doi:10.3390/v15040890)
Supplement: Supplementary file 1 [file viruses-15-00890-s001.zip › viruses-2198331-supplementary.pdf]

Table S1. Primers for (quantitative) PCR assay recommended by the National Standards of China

Viruses	Primers	Sequences (5′-3′)	Product size(bp)	No. of reference standard
CSFV	CSFV-qF	TACAGGACAGTCGTCAGTAGTTCGA-3′	/[a]	GB/T 27540-2011
	CSFV-qR	CCGCTAGGGTTAAGGTGTGTCT		
	CSFV-qP	FAM-CCCACCTCGAGATGCTATGTGGACGA-TAMRA		
ASFV	VP72-F1	GCTTTCAGGATAGAGATACAGCTCT	/[a]	GB/T 18648-2020
	VP72-R1	CCGTAGTGGAAGGGTATGTAAGAG		
	VP72-T1	FAM-CCGTAACTGCTCATGGTATCAATCTTATCG-BHQ1		
PRRSV	PRRSV-P_1	GGTTCGGAAGAAACTGTCGG	HP[b]: 400<br>LP[c]: 264	GB/T 27517-2011
	PRRSV-P_2	AGCAGGTGGAAGAAGCGAATC		
	PRRSV-P_3	GAGCTGAGTATTTTGGGCGTG		
PPV	PPV-F	TGGTCTCCTTCTGTGGTAGG	445	SN/T 1874-2007
	PPV-R	CAGAATCAGCAACCTCAC		
PRV	PRV-gD-P_1	CAGGAGGACGAGCTGGGGCT	217	GB/T 18641-2018
	PRV-gD-P_2	GTCCACGCCCCGCTTGAAGCT		
PCV	PCV-P_1	CCGCGGGCTGGCTGAACTT	PCV-1: 652<br>PCV-2: 1154	GB/T 21674-2008
	PCV-P_2	CTCGGCTATGCGCTCCAAAATG		
	PCV-P_3	ACCCCCGCCACCGCTACC		

[a] /: real time quantitative PCR; [b] HP: high pathogenic; [c] LP: low pathogenic
